# Incidence Rates and Predictors of Recurrent Long-Term Mental Sickness Absence Due to Common Mental Disorders

**DOI:** 10.1007/s10926-024-10226-7

**Published:** 2024-07-27

**Authors:** Matthew Mulder, Robin Kok, Bart Aben, Astrid de Wind

**Affiliations:** 1 Department of Research and Development, HumanTotalCare B.V., Zwarte Woud 10, Utrecht, 3524 SJ The Netherlands; 2 Amsterdam UMC Location University of Amsterdam, Public and Occupational Health, Amsterdam, The Netherlands; 3 Amsterdam Public Health Research Institute, Societal Participation & Health, Amsterdam, The Netherlands

**Keywords:** Cox proportional hazards model, Occupational health, Prognostic factor, Recurrent sickness absence, Sick leave, Survival analysis

## Abstract

**Purpose:**

Several predictors have been identified for mental sickness absence, but those for recurrences are not well-understood. This study assesses recurrence rates for long-term mental sickness absence (LTMSA) within subgroups of common mental disorders (CMDs) and identifies predictors of recurrent LTMSA.

**Methods:**

This historical prospective cohort study used routinely collected data from 16,310 employees obtained from a nationally operating Dutch occupational health service (ArboNed). Total follow-up duration was 23,334 person-years. Overall recurrence rates were assessed using Kaplan–Meier estimators. Recurrence rates within subgroups of CMDs were calculated using person-years. Univariable and multivariable Cox proportional hazards models were used to identify predictors.

**Results:**

15.6% of employees experienced a recurrent LTMSA episode within three years after fully returning to work after a previous LTMSA episode. Highest recurrence rates for LTMSA were observed after a previous LTMSA episode due to mood or anxiety disorders. Mood or anxiety disorders and shorter previous episode duration were predictors of recurrent LTMSA. No associations were found for age, gender, company size, full-time equivalent and job tenure.

**Conclusion:**

Employees should be monitored adequately after they fully returned to work after LTMSA. It is recommended to monitor high-risk employees (i.e. employees with mood or anxiety disorders and short LTMSA episode) more intensively, also beyond full return to work. Moreover, diagnosis of anxiety and depressive symptoms should be given a higher priority in occupational healthcare.

**Supplementary Information:**

The online version contains supplementary material available at 10.1007/s10926-024-10226-7.

## Introduction

Common mental disorders (CMDs) are the leading cause of sickness absence (SA) in most Western countries [1, 2]. In the Netherlands, the proportion of long-term mental sickness absence (*LTMSA*; i.e. > 42 days) has rapidly increased over the past years. In 2015, mental disorders accounted for 26% of long-term SA causes and this increased annually up to 40% in 2020. Concomitantly, the mean absence duration rose in this five-year period [3], incurring high expenses for employers. SA due to stress symptoms or adjustment disorders such as burnout and overexertion is estimated to cost Dutch employers nearly 20,000 euros per sick employee [4]. Mental illness is the main cause for disability benefit applications in the Netherlands and is associated with a lower probability of return to work (RTW) after the benefit is granted in comparison to physical disorders [5]. Moreover, mental sickness absence (MSA) involves a high burden of disease for employees.

To prevent MSA, it is important to identify risk factors at an early stage. So far, various psychosocial, medical, organizational and work-related predictors of MSA have been suggested [6, 7]. A recent scoping review showed that female gender, lower educational level, previous episodes of mental disorders, higher symptom severity, comorbidity, lower perceived general health, smoking, low job control, high job demands and high job strain were risk factors for MSA in employees having a CMD [8].

Recurrent MSA often occurs within two years after employees have fully returned to work [9–13]. Several studies also show longer recovery times for recurrent MSA episodes [9–11], which therefore have a larger socio-economic impact than previous episodes of MSA. Previous work has indicated that higher recurrence rates are observed in employees with a MSA episode due to depressive disorders compared to those with adjustment disorders and anxiety disorders [10, 13]. However, these studies did not reveal which specific CMDs appeared as recurrent MSA episodes, leading to the question whether it is likely that the same CMD reappears, or whether psychological symptoms manifest themselves as a different disorder. With more insight into the mechanisms of recurrent MSA, occupational physicians would be better able to monitor and counsel employees who are at higher risk of recurrent MSA.

Several attempts have been made to identify predictors of recurrent MSA but the evidence is mixed. A recent scoping review [8] demonstrated that previous SA and shorter job tenure were the most consistent predictors of recurrent MSA. Gender [9, 12–17] and full-time employment [9, 12, 13] were no predictors, whereas the predictive value of age was unclear due to conflicting results in studies [9, 12–17]. One study suggested that larger company size (> 100 employees) was associated with recurrent MSA [18], but evidence is uncertain as this finding has not yet been replicated.

Altogether, it is clear that recurrent MSA has major personal and socioeconomic consequences, but recurrence rates and predictors are not well established. In order to prevent recurrent MSA, recurrence rates and robust predictors need to be identified. The present study focused on LTMSA as this incurs the highest burden of disease for employees and the highest socio-economic costs. This study aimed to determine recurrence rates for LTMSA within different groups of CMDs and identify predictors of recurrent LTMSA.

## Methods

### Study Setting and Population

This was a historical prospective cohort study. Routinely collected data from ArboNed, a Dutch national occupational health service (OHS), were analysed. ArboNed provides occupational healthcare to more than 650,000 employees of over 60,000 contracted companies, of which approximately 75% are small and medium-sized enterprises (up to 250 employees) [19]. Data consisted of demographic characteristics of employees of the companies served by the OHS, including age and gender. These data were supplemented with data of SA and work-related variables. As required by law, data on SA are provided by the employer of a sick employee through a standardized and automated digital registration process. The dataset was fully anonymized before it was made available to the researchers. As the data were routinely collected as part of the OHS's care provision, approval of a medical ethics committee was not necessary.

This study included employees having a LTMSA episode due to a CMD, i.e. adjustment disorder, mood disorder, anxiety disorder or post-traumatic stress disorder (PTSD). PTSD was included as a CMD, because comorbid depressive or anxiety disorders are common [20]. A few diagnoses were included in a’residual CMD group’ as they did not completely meet definitions to fit in other CMD groups. More details on specific diagnoses included in this study are provided in the supplemental table.

Other inclusion criteria were age between 18 and 66 years, the latter being the state pension age in the Netherlands during the period of data collection (i.e. 1 January 2015 until 23 November 2022; [21]), and employment for at least 0.1 full-time equivalent (fte).

After applying inclusion criteria and definitions of (recurrent) LTMSA (see below), 16,909 employees were eligible for inclusion in this study. A total of 332 employees (2.0%) were removed from the database due to administrative errors (e.g. the employee was aged 30 on the first day of the index episode and had a job tenure of 20 years). Afterwards, 267 out of the remaining 16,577 employees (1.6%) were excluded for analyses due to missing data on one or more variables. Subsequently, 16,310 employees were included in this study.

### Definitions of (Recurrent) LTMSA

**Fig. 1:**
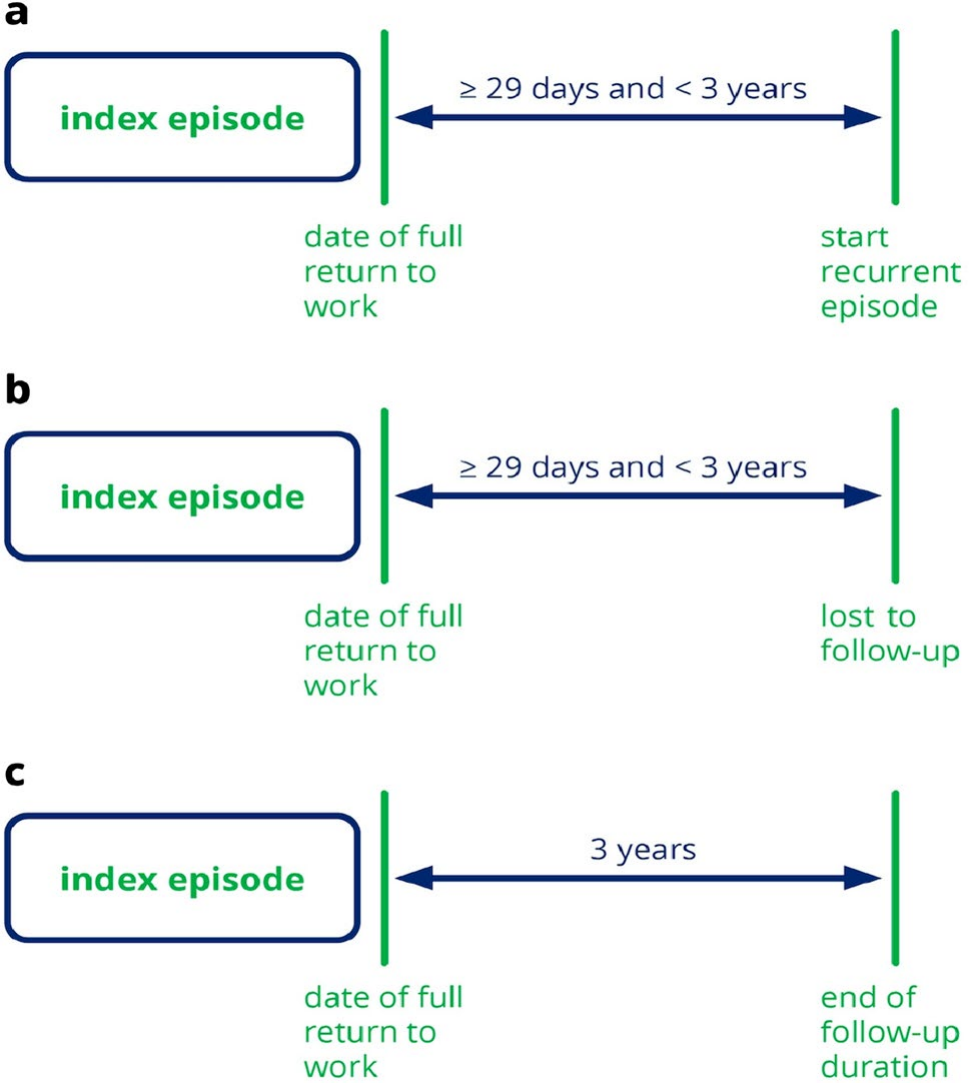
Follow-up possibilities within the period of data collection (1 January 2015 until 23 November 2022) of this historical prospective cohort study

The current study assessed index episodes as the first LTMSA episode due to a CMD during the observation period. A subsequent LTMSA episode was considered as a recurrent episode. LTMSA was defined as > 42 consecutive days of SA, as in the Netherlands occupational physicians must assess work capacity and assign a formal diagnosis to the SA episode within the first 42 days of SA [19]. Maximum duration of index episodes was set to two years, after which employees can apply for disability benefits [5] and are lost to follow-up to the occupational health service. An index episode was defined as the first episode of LTMSA since 1 January 2015, the earliest date for which data were available. A recurrent episode was defined as the first subsequent episode of LTMSA after an index episode. The first absence day of recurrent LTMSA had to be between 29 days and three years after full RTW, as Dutch SA insurance policies stipulate that SA within four weeks after full RTW is a continuation of the previous SA episode [9, 13, 15]. Literature shows that 76–90% of recurrences start within three years after index episodes have ended [9, 13], indicating that opting for a three-year follow-up duration suffices to detect the vast majority of recurrences. Follow-up ended when a recurrent episode occurred (Fig. 1a), an employee was lost to follow-up (Fig. 1b) or when the maximum follow-up time of three years was exceeded without the occurrence of a recurrent episode (Fig. 1c). In the event that a recurrent LTMSA episode occurred, employees were treated as uncensored, whereas employees that did not experience a recurrence within the follow-up period were treated as right-censored.

### Variables

Data included demographic variables (i.e. age and gender), disease-related variables (i.e. index CMD group and index episode duration) and work-related variables (i.e. company size, fte, job tenure and employment sector).

Age, index episode duration, company size, fte and job tenure were analysed as continuous variables, whereas gender and index CMD group were analysed as categorical variables. These variables were considered as potential predictors of recurrent LTMSA. Employment sector was only used to describe the study population. Age, company size, fte and job tenure were based on the data at the first absence day of the index episode. Company size on the last absence day of the index episode was used in 379 out of 16,310 included cases (2.3%), as this information was missing for the first absence day. One fte varies between 36 and 40 h, depending on the Collective Labour Agreement. Standard Industrial Classification codes for economic activities were followed to define employment sectors [22].

### Statistical Analyses

All statistical analyses were performed using IBM SPSS Statistics (Version 28.0). Figures were created with the lifelines packages (0.26.3) in Python (Version 3.8). Descriptive statistics were used to summarize the study population.

Overall recurrence rate for LTMSA was obtained through Kaplan–Meier estimation. Incidence rates (IRs) for recurrent LTMSA within different CMD groups were calculated using person-years of follow-up duration to account for censored data. These were expressed per 1,000 person-years to enhance comparability. For instance, an IR of 10.0 per 1,000 person-years would mean that in one year, ten out of 1,000 employees have a recurrent LTMSA episode.

Cox proportional hazards (CPH) regression models were used to identify predictors for recurrent LTMSA. For categorical variables (i.e. gender and index CMD group) the largest group was considered as the reference group. Multicollinearity between variables was assessed through variance inflation factors. All eight variables had scores < 5, which indicated they were only (very) weakly correlated [23]. Proportional hazards assumptions were tested by adding terms for time by variable interactions and assessing their statistical significance (alpha = 0.05). Gender and index episode duration showed a significant interaction with time in univariable and multivariable analyses. Further inspection of stratified Kaplan–Meier curves and plots of Schoenfeld residuals for the effect of gender did not show a clear (linear) relationship between hazard ratio and time. The average Schoenfeld residual rather oscillates around 0. To avoid overfitting, it was decided not to include the time by gender interaction in the models. The Schoenfeld residuals for index episode duration did show a linear upward trend. Therefore, the interaction term for time by index episode duration was included in the models. All variables were added simultaneously to the multivariable model.

Univariable and multivariable CPH analyses were performed to calculate hazard ratios (HRs) and their 95% confidence intervals (CIs). In both analyses, variables were considered as significantly associated with recurrent LTMSA if *p* < 0.05. Hazard ratios for index episode duration were computed at different time points (0, 365, 730 and 1095 days).

## Results

### Descriptives of Study Population

Of the 16,310 employees included in this study (Table 1), 7,887 were female (48.4%). Median age was 41 years [interquartile range (IQR): 33–50]. The index episode concerned predominantly adjustment disorders (64.0%). Mood disorders, anxiety disorders, PTSD and residual CMDs accounted for 19.9%, 9.8%, 4.5% and 1.9%, respectively. The vast majority of mood disorders were depression (1,505 of 1,591 cases). Median index episode duration was 171 days (IQR: 102–279), median company size was 89 employees (IQR: 32–220), median fte was 1.0 (IQR: 0.8–1.0) and median job tenure was 7.6 years (IQR: 3.1–13.9). The most common employment sector was ‘wholesale and retail trade; repair of motor vehicles and motorcycles’, followed by ‘manufacturing’ and ‘professional, scientific and technical activities’. These three sectors accounted for approximately half of all employees.

**Table 1:** Baseline characteristics of employees (*N* = 16,310)

Variable	*n*	%	Median	IQR	Range
Age (years)			41	33–50	18–66
Gender					
Female	7,887	48.4			
Male	8,423	51.6			
Index CMD group^a^					
Adjustment disorders	10,431	64.0			
Mood disorders	1,591	9.8			
Anxiety disorders	726	4.5			
PTSD	318	1.9			
Residual CMDs	3,244	19.9			
Index episode duration (days)			171	102–279	43–729
Company size			89	32–220	1–3,528
Fte			1.0	0.8–1.0	0.1–1.0
Job tenure (years)			7.6	3.1–13.9	0.0–48.2
Employment sector^b^					
A: Agriculture, forestry and fishing	24	0.1			
B: Mining and quarrying	26	0.2			
C: Manufacturing	2,341	14.4			
D: Electricity, gas, steam and air conditioning supply	23	0.1			
E: Water supply; sewerage, waste Management and remediation activities	109	0.7			
F: Construction	957	5.9			
G: Wholesale and retail trade; repair of motor vehicles and motorcycles	3,486	21.4			
H: Transportation and storage	576	3.5			
I: Accommodation and food service activities	206	1.3			
J: Information and communication	1,085	6.7			
K: Financial and insurance activities	1,096	6.7			
L: Real estate activities	262	1.6			
M: Professional, scientific and technical activities	2,164	13.3			
N: Administrative and support service activities	911	5.6			
O: Public administration and defence; compulsory social security	131	0.8			
P: Education	489	3.0			
Q: Human health and social work activities	1,345	8.2			
R: Arts, entertainment and recreation	404	2.5			
S: Other service activities	675	4.1			
T: Activities of households as employers; undifferentiated goods-and services-producing activities of households for own use	0	0.0			
U: Activities of extraterritorial organizations and bodies	0	0.0			

*CMD*, common mental disorder, *fte* full-time equivalent, *IQR* interquartile range, *PTSD* post-traumatic stress disorder.

^a^The distribution of more specific diagnoses within the CMD groups with corresponding percentages is provided in the supplemental table

^b^In total, 16,310 employees were divided over 3,657 different employers

### Kaplan–Meier Estimates for Recurrent LTMSA Due to CMDs

**Fig. 2:**
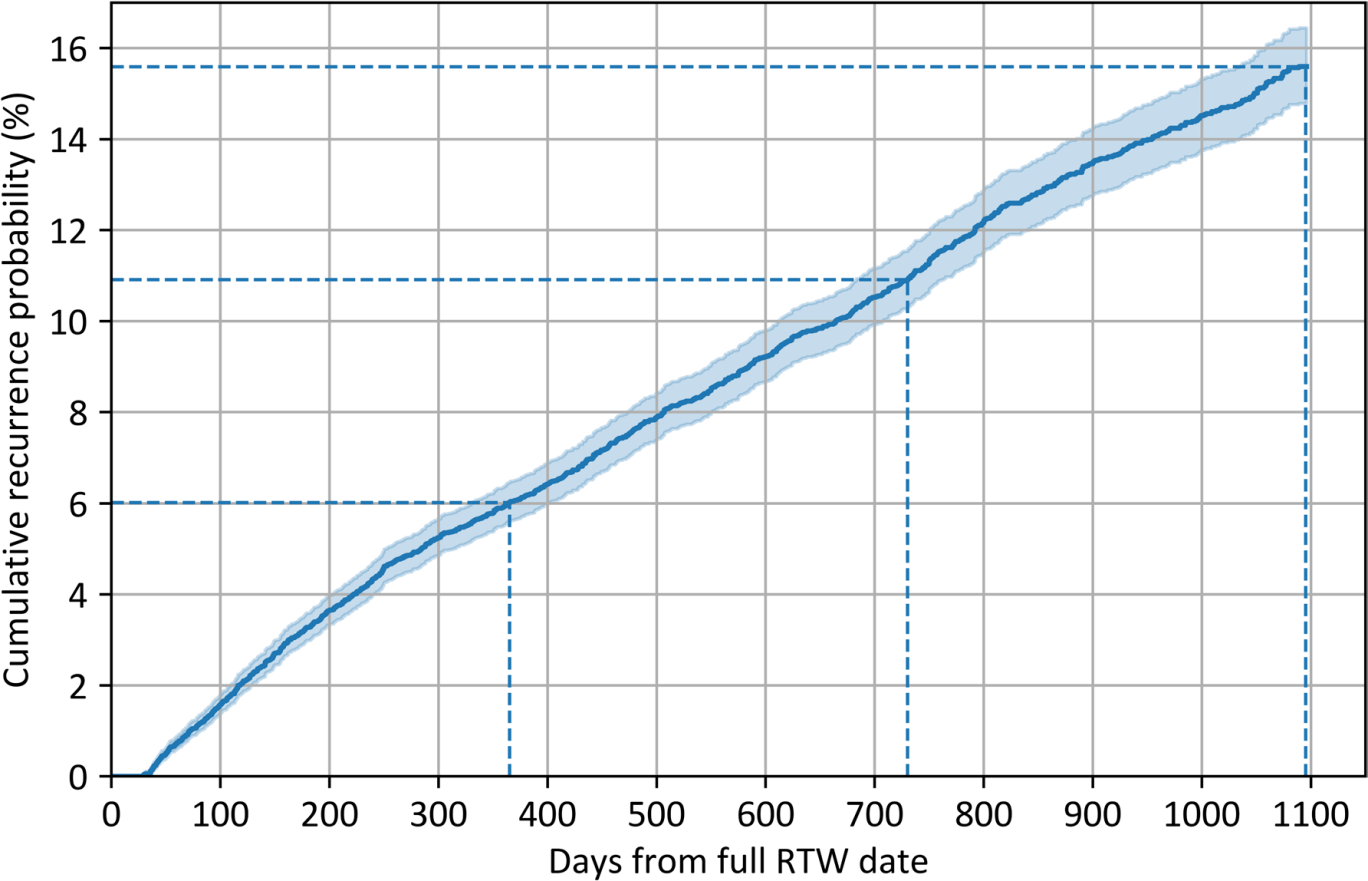
Kaplan–Meier curve for cumulative probability of recurrent long-term mental sickness absence. *N* = 16,310 employees. Error bands represent 95% confidence intervals. A recurrent episode of long-term mental sickness absence (LTMSA) occurred in 6.0% (95% CI: 5.6–6.5) of employees within one year, in 10.9% (95% CI: 10.3–11.6) within two years and in 15.6% (95% CI: 14.8–16.4) within three years after a previous LTMSA episode followed by full return to work (RTW)

The Kaplan–Meier recurrence curve (Fig. 2) shows that 6.0% of employees (95% CI: 5.6–6.5) have a recurrent LTMSA episode within one year, 10.9% (95% CI: 10.3–11.6) within two years and 15.6% (95% CI: 14.8–16.4) within three years after a previous LTMSA episode followed by full RTW. Median time to recurrence was 317 days (IQR: 137–618) and median follow-up time in employees with no recurrent episode was 436 days (IQR: 138–1034).

### Incidence Rates for Recurrent LTMSA Within CMD Groups

Table 2 shows IRs of recurrent LTMSA within CMD groups. The recurrence rate was 58.2 per 1,000 person-years (95% CI: 55.1–61.2) after any CMD group as index episode, which meant that 58.2 per 1,000 employees per year had a recurrent LTMSA episode. Most recurrences emerged after an index episode of anxiety disorders (75.8 per 1,000 person-years, 95% CI: 59.2–92.5), whereas adjustment disorders resulted in fewer recurrences (54.3 per 1,000 person-years, 95% CI: 50.5–58.0) compared to other index CMD groups.

**Table 2:** Incidence rates of recurrent long-term mental sickness absence (LTMSA), stratified by CMD groups. *N* = 16,310 employees

	CMD group recurrent episode, IR (95% CI)					
	Adjustment disorders, *n* = 807	Mood disorders, *n* = 161	Anxiety disorders, *n* = 80	PTSD, *n* = 30	Residual CMDs, *n* = 279	All recurrent episodes, n = 1,357
CMD group index episode (person-years at risk)						
Adjustment disorders (14,869 years)	35.2 (32.2–38.3)	6.1 (4.9–7.4)	3.3 (2.4–4.2)	1.3 (0.8–1.9)	8.3 (6.8–9.7)	54.3 (50.5–58.0)
Mood disorders (2,338 years)	22.7 (16.6–28.8)	34.6 (27.1–42.2)	3.8 (1.3–6.4)	1.3 (0.0–2.7)	6.4 (3.2–9.7)	68.9 (58.2–79.5)
Anxiety disorders (1,055 years)	19.0 (10.6–27.3)	11.4 (4.9–17.8)	36.0 (24.6–47.5)	1.9 (0.0–4.5)	7.6 (2.3–12.8)	75.8 (59.2–92.5)
PTSD (435 years)	25.3 (10.4–40.3)	2.3 (0.0–6.8)	4.6 (0.0–11.0)	29.9 (13.7–46.2)	2.8 (0.0–14.7)	65.0 (44.3–93.7)
Residual CMDs (4,637 years)	26.3 (21.6–31.0)	7.1 (4.7–9.5)	3.7 (1.9–5.4)	0.9 (0.0–1.7)	22.2 (17.9–26.5)	60.2 (53.1–67.2)
All groups (23,334 years)						58.2 (55.1–61.2)

All incidence rates are expressed per 1,000 person-years

*CI* confidence interval, *CMD* common mental disorder, *IR* incidence rate, *PTSD* post-traumatic stress disorder.

Index episodes were usually followed by recurrent episodes from the same diagnostic CMD group (e.g. adjustment disorders were most commonly followed by adjustment disorders). Contrarily, adjustment disorders emerged as the most common recurrent LTMSA episode after a residual CMD as index episode. Adjustment disorders were consistently one of the two most common recurrent CMD groups, irrespective of the CMD group as index episode.

### CPH models for predictors of recurrent LTMSA

Table 3 shows HRs of variables in univariable and multivariable CPH analyses. In both univariable and multivariable analyses, age, gender, company size and fte did not predict recurrent LTMSA, whereas index CMD group and index episode duration did. Job tenure was only predictive in univariable analysis.

**Table 3:** Cox proportional hazards analyses

	Univariable analysis (N = 16,310)			Multivariable analysis (N = 16,310)		
Variable	p-value	HR	95% CI	p-value	HR	95% CI
Age (in years)	0.070	0.995	0.990–1.000	0.758	0.999	0.993–1.005
Gender						
Male (ref)						
Female	0.309	0.946	0.850–1.053	0.689	0.976	0.866–1.100
Index CMD group	0.004*	N/A	N/A	0.004*	N/A	N/A
Adjustment disorder (ref)						
Mood disorder	0.006*	1.271	1.073–1.505	0.004*	1.281	1.080–1.518
Anxiety disorder	0.004*	1.397	1.110–1.758	0.006*	1.387	1.101–1.748
PTSD	0.199	1.270	0.882–1.828	0.176	1.287	0.893–1.855
Residual CMDs	0.133	1.110	0.969–1.272	0.215	1.091	0.951–1.251
Index episode duration (in months; *t* = 0 days)	< 0.001*	0.958	0.938–0.978	< 0.001*	0.957	0.937–0.978
Index episode duration (t = 365 days)		0.982	0.961–1.003		0.981	0.969–0.994
Index episode duration (t = 730 days)		1.007	0.985–1.028		1.006	0.988–1.024
Index episode duration (t = 1095 days)		1.032	1.010–1.054		1.031	1.000–1.063
Company size (per hundred employees)	0.256	1.012	0.991–1.034	0.328	1.011	0.989–1.032
Fte (per 0.1)	0.097	1.027	0.995–1.060	0.155	1.026	0.990–1.062
Job tenure (in years)	0.030*	0.993	0.986–0.999	0.121	0.994	0.986–1.002

*CI* Confidence interval, *CMD* Common mental disorder, *fte* Full-time equivalent, *HR* Hazard ratio, *N/A* Not applicable, *PTSD* Post-traumatic stress disorder, *ref*, Reference group

*Significant *p*-value

In multivariable analyses, employees with mood disorders and anxiety disorders had, respectively, a 28.1% (8.0–51.8%; *p* = 0.004) and a 38.7% (95% CI: 10.1–74.8, *p* = 0.006) higher recurrence rate compared to employees with adjustment disorders. No other significant comparisons between index CMD groups were found. Each additional month of index episode duration reduced the recurrence rate by 4.3% (95% CI: 2.2%-6.3%; *p* < 0.001) at *t* = 0 days. As time since RTW progressed, the effect of index episode duration attenuated; after 730 days, the risk of recurrent LTMSA converged on a HR of 1.

## Discussion

The present study shows that mood or anxiety disorders (compared to adjustment disorders) and shorter index episode duration are associated with a higher risk of recurrent LTMSA due to CMDs. The association for index episode duration attenuates as time since RTW progresses. There is no indication that age, gender, company size, fte and job tenure are associated with recurrent LTMSA.

In line with smaller-scale previous studies [10, 13], this study shows higher recurrence rates for depressive disorders compared to adjustment disorders. In contrast to these studies, the highest recurrence rate in the current study is found for anxiety disorders as index CMD group. This is in line with epidemiological findings from the general population [24]. Additionally, the present study shows which specific CMD groups appeared more frequently as recurrent LTMSA episodes. Employees with any previous LTMSA episode are particularly at higher risk for future LTMSA due to an adjustment disorder or a diagnosis belonging to the same CMD group as the previous LTMSA episode.

Differences with previous research were mainly observed for the predictive value of index CMD groups and index episode duration. Real et al. [17] showed that index diagnosis did not predict recurrent MSA, but their CMD groups also included substance dependence and psychotic, affective and personality disorders, and therefore did not match the data of the current study.

The present study shows that longer index episode duration was associated with a lower risk of recurrent LTMSA in the short-term (e.g. < 1 year), but that this effect attenuates as time since RTW progresses. It is plausible that employees who take more time to fully recover from a CMD have a decreased risk of early recurrence (e.g. in the first year after recovery). In the long run, however, the length of the index episode is no longer a predictive factor. Previous studies [14, 16] found no association between index episode duration and recurrent LTMSA.

### Strengths and Limitations

The present study has several strengths. First, a large sample of employees (N = 16,310) distributed across many employment sectors was included, using register data rather than self-reported data for absence duration. Second, data were routinely collected and the risk of selection bias is therefore low. Third, the low quantity of missing data makes it unlikely that exclusion of corresponding employees introduced bias in outcomes. Fourth, unlike previous studies, continuously distributed variables were not categorized, which had the aim of maintaining as much variance as possible and thus not losing power [25].

One limitation is that from the moment an employee moves to another employer, the employee is lost to follow-up, and a recurrent episode may have occurred outside of the data collection period. Recurrence rates could have been underestimated if workers with higher recurrence risk were more likely to change jobs. The fact that the predictors index episode duration and job tenure are time-dependent variables could have introduced a bias in the model. For example, in cases with a long index episode duration, a correspondingly extended window for data collection is needed to encompass three years of follow-up. In other words, a long index episode duration may increase the probability of loss-to-follow-up and hence the chance that a recurrent LTMSA occurred outside the period of data collection. Indeed, employees lost to follow-up exhibited a longer median index absence duration. Nevertheless, mitigating this association through the reduction of the follow-up period to one year did not affect the association between index episode duration and recurrent LTMSA, arguing against presumption of bias. We performed similar checks for job tenure and found no signs of bias for this predictor either.

Lastly, a limitation that is common to routinely collected data is that it is rarely exhaustive. An employee may have had a LTMSA episode before the index episode, so an index episode in the current study could also be a recurrence. However, a subsequent episode is always a recurrence, regardless of whether it is the first or one of many.

### Practical Implications and Further Research

As more than one out of seven employees have a recurrent LTMSA episode within three years after full RTW, and as the median time to recurrence is less than one year, it may be beneficial to monitor employees for at least one year after they have fully returned to work. Monitoring after RTW is not a major part of the Dutch occupational health guideline for mental problems (2019), which states that guidance by an occupational physician can be ended after full RTW if the employee has recovered to optimal functioning, and if this can be sufficiently guaranteed [26]. High-risk employees, such as employees with (comorbid) anxiety or depressive disorders, should be monitored more intensively.

Monitoring is possible with questionnaires or occupational health consultations and should have a focus on the development of mental symptoms over the past weeks. By this way of monitoring, occupational physicians will be able to counsel on time (e.g. recommend to start a psychological intervention). Previous research has already shown effectiveness of preventive actions after MSA [18]. Employers could also play a role in monitoring employees after they fully returned to work, either internally or in collaboration with occupational health services. Employers have a strong interest in preventing a recurrent LTMSA episode, as this incurs significant financial consequences [4]. They can play a vital role in preventing recurrent LTMSA by providing workplace accommodations, such as the ability to modify job tasks and scheduling working hours more flexible [27]. Employers could engage in dialogue with their employees to identify their needs for workplace accommodations, so that these can be practically implemented with the aim of ensuring sustainable employability.

In current occupational healthcare practice, much attention is paid to burnout and overexertion, presumably because of ever increasing workloads. This focus on adjustment disorders may lead to an underreporting of anxiety and depressive disorders. Moreover, comorbid anxiety or depressive symptoms in adjustment disorders are common and psychiatric comorbidity negatively impacts recurrence rate [28]. Increase in comorbid symptoms could lead to the development of anxiety and depressive disorders as main diagnoses, which is not desirable due to their higher risks on recurrences. IRs in literature support that there appears to be a hyperfocus on adjustment disorders in occupational healthcare practice. Anxiety and depressive disorders emerge more often than adjustment disorders in the general population [29, 30], whereas almost two third of index diagnoses were adjustment disorders in the present study. Altogether, more attention for (comorbid) anxiety and depressive symptoms is urgently needed, as such low prevalence of anxiety and depressive disorders in occupational health is unlikely.

Future research should further investigate which work-related and particularly non-work-related factors (such as providing informal care and cohabiting) are associated with recurrent MSA. So far, many factors have not been examined in previous research or have solely been examined in a few studies. Future studies are therefore needed to better understand which workers are at high risk of recurrent MSA in order to determine which type of employees should be monitored more intensively or for a longer period of time after a previous episode of MSA. Prevention of future MSA could save suffering and costs.

One relevant factor to further investigate is frequent short-term SA, because previous studies have already shown that it predicts future long-term SA [31–34]. Moreover, a combination of frequent short-term SA and long-term SA resulted in a higher risk of future long-term SA than when only one of these factors was present [32]. All types of SA were included in that study, and it is relevant to know whether this also specifically applies to MSA, so that high-risk employees can be identified.

Another factor on which more knowledge should be gathered is somatic comorbidity as this is common in CMDs and, furthermore, as a higher number of somatic comorbidities is related to a higher prevalence of CMDs [35]. Although comorbidity has already been examined in previous studies about recurrent MSA, its predictive value remains uncertain because results are conflicting [10, 11, 13, 18].

## Conclusion

This study has focused on recurrent LTMSA due to CMDs in predominantly small and medium-sized enterprises and has shown that adjustment disorders yield lower recurrence rates compared to mood or anxiety disorder. Paradoxically, adjustment disorders are highly prevalent as recurrent episodes after any type of index episode. These findings indicate that there appears to be a hyperfocus on adjustment disorders in occupational healthcare practice. In the light of current findings, occupational physicians should be aware of frequently occurring comorbid anxiety and depression.

This study also shows that employees with a previous LTMSA episode due to a mood or anxiety disorder (compared to adjustment disorders) and employees with a shorter previous episode duration are at higher risk of recurrent LTMSA. The effect of previous episode duration attenuates as time since RTW progresses. Since recurrent LTMSA due to CMDs is common, it is recommended to monitor employees for at least one year after full RTW and to counsel when needed.

## Supplementary Information

Below is the link to the electronic supplementary material.

Supplementary file1 (DOCX 16 KB)

## Data Availability

No datasets were generated or analysed during the current study.
