# Supplementary material for: Incidence Rates and Predictors of Recurrent Long-Term Mental Sickness Absence Due to Common Mental Disorders
Source: J Occup Rehabil. 2024 Jul 27;35(3):592–601. doi: 10.1007/s10926-024-10226-7 (PMC12361271; doi:10.1007/s10926-024-10226-7)
Supplement: Supplementary file 1 — Supplementary file1 (DOCX 16 KB) [file 10926_2024_10226_MOESM1_ESM.docx]

**Supplementary material**

**Supplemental table** The distribution of classification codes for occupational and social insurances (CAS codes) within the CMD groups (N=16,310 employees). In the Netherlands, occupational physicians classify diagnoses with CAS codes, which are derivatives of the International Classification of Diseases codes.

| **Index CMD group (CAS code)** | | **n (%)** | |
| --- | --- | --- | --- |
| Adjustment disorders | | 10,431 (64.0) | |
|  | Burnout (P611) |  | 1,933 (11.9) |
|  | Other adjustment disorders or overexertion (P619) |  | 8,498 (52.1) |
| Mood disorders | | 1,591 (9.8) | |
|  | Depressive disorder (P652) |  | 1,505 (9.2) |
|  | Bipolar affective disorder (P651) |  | 41 (0.3) |
|  | Other mood disorders (P659) |  | 45 (0.3) |
| Anxiety disorders | | 726 (4.5) | |
|  | Panic disorder (P630) |  | 302 (1.9) |
|  | Generalized anxiety disorder (P631) |  | 111 (0.7) |
|  | Agoraphobia (P632) |  | 2 (<0.1) |
|  | Social phobia (P633) |  | 2 (<0.1) |
|  | Obsessive-compulsive disorder (P692) |  | 38 (0.2) |
|  | Other anxiety disorders (P639) |  | 271 (1.7) |
| PTSD (P620) | | 318 (1.9) | |
| Residual CMDs | | 3,244 (19.9) | |
|  | Tension symptoms / agitation and anger / nervous tension / rumination (P109) |  | 2,828 (17.3) |
|  | Other reactions on severe stress (P629) |  | 416 (2.6) |

Abbreviations: CMD, common mental disorder; PTSD, post-traumatic stress disorder.
